# Real-world studies: bridging the gap between trial-assessed efficacy and routine care

**DOI:** 10.7555/JBR.36.20220040

**Published:** 2022-05-10

**Authors:** Daohong Chen

**Affiliations:** Research Institute, Changshan Biochemical Pharmaceutical, Shijiazhuang, Hebei 050800, China

**Keywords:** real-world evidence, therapeutic effectiveness, pharmacovigilance

## Abstract

Even though randomized controlled clinical trials (RCTs) have been accepted as the gold standard for official assessment of novel interventions, there is a substantial gap between the efficacy observed in RCTs and the impact on clinical practice and in terms of patient benefit. While real-world studies (RWS) are emerging to confer valuable complementing evidence in this regard and beyond, the evolving role of RWS is yet to be agreed. This article delineates an updated profile of RWS covering effectiveness verification, rare adverse effects discovery, indication repurposing, to name a few. RWS tends not only to improve the efficiency of clinical investigations for regulatory approval, but also optimizes the whole-life cycle evaluation of biomedical/pharmaceutical products.

## Introduction

Randomized controlled trials (RCTs) play an indispensable role in assessing the efficacy and safety of therapeutic agents prior to marketing for human use^[1–2]^. In order to minimize bias, RCT protocols stipulate the type of processing required to distinguish the efficacy of an intervention from routine practice. As such, protocols include comprehensive eligibility criteria to which are often designed to exclude those with various co-morbidities and particular sub-groups because these factors may confound findings^[3]^. There are also always economic constraints which ensure sample sizes and the duration of RCTs are considered to fulfill basic statistical requirements. However, this may not be enough to identify rare events or long-term effects associated with the intervention of interest^[4].^ These shortcomings have been widely discussed and are considered serious issues which need to be addressed^[3–4]^.

Real-world studies (RWS) have emerged as post-market surveillance approach to assess performance of existing drugs. RWS tend to supplement RCTs, in particular of those with accelerated regulatory approval^[3,5]^. RCTs are traditionally designed in prospective modes with an independent trial master file and case report forms, which are separated from routine medical care records^[1]^. By contrast, RWS can be either retrospective or/and prospective, and have more efficient quality controls. RWS also have the potential to observe larger patient populations across longer timelines which is particularly useful for monitoring rare adverse events as well as recurring effects^[3,6]^. The term RWS is an umbrella for, real-world evidence (RWE) which is extracted from screening, and real-world data (RWD) which can be collected from a number of sources including electronic health records (EHR), paper medical records, professional patient data banks, publications, and even medical claims/billing data, *etc*^[4,7]^. By integrating an RWS approach into different contexts researchers are able to lower relative research costs while exploring the therapeutic effects of interventions in everyday life^[4]^.

**Figure 1:**
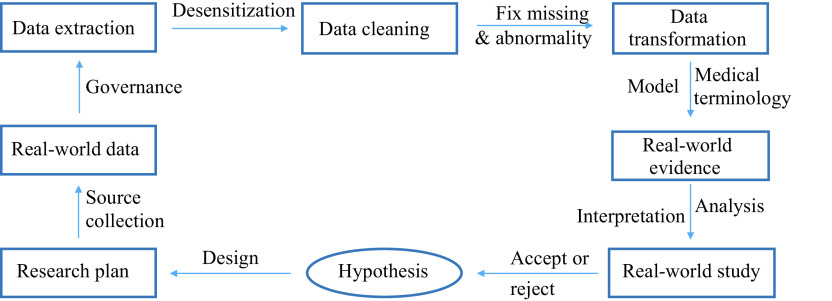
The processing flow chart of real-world study.

Having been initially introduced to accelerate novel therapeutics development by the United States (US) congress in 2016, RWE was further proposed to support clinical indication expansion of approved drugs and to address certain post-approval requirements established by US Food and Drug Administration (FDA) in 2018^[7–8]^ . The concept of RWE has interested a number of international regulatory agencies and clinical investigators including those in Europe and China^[4,9–11]^. Indeed, RWE-based RWS are increasingly being conducted to investigate biomedical challenges, in particular during the coronavirus disease 2019 (COVID-19) pandemic. This is because of the nature of vaccine trials and the demand for rapid approval; however, the exact role of RWS in pharmaceutical innovations is yet to be determined^[7,12]^. This article considers the evolving purpose of RWS in the changing landscape bridging the whole life cycle of drug assessment and into common use (***Table 1***, ***Fig. 1***).

**Table 1:** Emerging roles of real-world study

Goal	Applications	Examples discussed	References
Regulatory relevance	Effectiveness corroboration	Remdesivir	[13–14]
		Linagliptin	[15]
		Pembrolizumab	[16]
	Rare adverse events	ICI	[6]
		SGLT-2 inhibitor	[17]
		Rofecoxib	[18]
	Indication repurpose	LMWH	[19–20]
		GLP-1 RA	[21]
		JAK-STAT inhibitor	[22]
Regulatory irrelevance	Biomarker validation	ctDNA	[23]
		BNP	[24]
		PCT	[25]
	Pharmacoeconomics	Liraglutide	[26]
		Abacavir	[27]
		Rituximab	[28]
	Clinical epidemiology	COVID-19 comorbidity	[29–30]
		COVID-19 test timing	[31]
		COVID-19 DDI	[32]
ICI: immune checkpoint inhibitor; SGLT: sodium glucose co-transporter; LMWH: low molecular weight heparin; GLP-1RA: glucagon-like peptide-1 receptor agonist; JAK-STAT: Janus kinases/signal transducer and activator of transcription; ctDNA: circulating tumor DNA; BNP: B-type natriuretic peptide; PCT: procalcitonin; COVID-19: coronavirus disease 2019; DDI: drug-drug interaction.			

## Effectiveness corroboration

New drug applications are typically approved following a period of validation which includes toxicity testing, safety, and efficacy which are determined through three main RCT phases. Phase Ⅲ studies are generally conducted with rather homogeneous populations for a limited period of time, and do not address the complexities of routine or individualized care^[3]^. The RWS approach, has complementary strengths which can generate additional insights into intervention's effectiveness and for generalizing findings to a broader population or indeed withdraw therapeutics from the market space. Intriguingly, there may be a niche for RWS researchers, given that RCTs only involve what are considered statistically necessary samples and are designed for a set time. Additionally, RCTs are not always an appropriate format for end-of-life studies because it would be unethical to establish a control group if a novel therapy has potential benefit^[12]^. The medical basis used to support switching between multiple biosimilars is also yet to be established while individual analogous products have achieved bioequivalence to originators. Therefore, the RWS approach becomes an attractive option to overcome the shortcomings involved in establishes phases of clinical trials^[33]^.

In the war against COVID-19, remdesivir appeared as a promising intervention with clear *in* *vitro* potency, inhibiting the viral RNA polymerase^[14]^. Remdesivir was consequently authorized for emergency use by FDA^[13]^. By way of a hybridized clinical trial which consisted of an RCT based remdesivir treatment group and a retrospective RWE cohort-control group researchers found that Remdesvir significantly improved recovery (74.4% *vs.* 59.0%) and decreased mortality at two weeks (7.6% *vs.* 12.5%)^[13]^. As well as ensuring there was a necessary comparator group of patients struggling to survive, this adaptive RWE approach provided evidence which is consistent with other randomized adaptive trials^[34]^.

Similarly, even though the therapeutic effects of dipeptidyl peptidase-4 (DPP-4) inhibitors on type 2 diabetes mellitus are understood, their effectiveness across sub-groups of age, ethnicities and renal function remains undefined. Through a retrospective RWS based adjusted analysis of EHRs, the DPP-4 inhibitor, Linagliptin, appears to result in a greater reduction of glycated hemoglobin in specific sub-populations. For example, older people, African Americans and those with higher estimated glomerular filtration rate appear to respond better to Linagliptin treatment^[15]^.

Additionally, given that antibody medicine has emerged as therapeutic means against major human diseases, relevant biosimilars are also rapidly emerging and include those which target tumor necrosis factor-α and interleukine (IL)-17^[33,35]^. Fortunately, an range of RCTs and RWS has been conducted to investigate whether these biosimilars are capable of replacing their originators^[33]^. RWS are also considered to play an important role in post-approval optimization and regimen frequencies for antibody drugs. For example, it is possible to determine extended dose intervals for immune checkpoint inhibitors which have similar outcomes to those receiving treatment at the label specified rate of three week intervals^[16]^.

## Rare or emerging adverse events

Aside from efficacy, safety profiles must be assessed through clinical studies before regulatory decisions are made for any therapeutic product. According to the principles of RCT design we are supposed to administer interventions to target patient population which excludes those with complex histories, co-morbidities, and the trial duration is acceptably limited to include major endpoints^[3,5]^. However in reality, numerous mainstream medications are prescribed for a life-time across much larger and more heterogeneous populations with comorbid diseases such as diabetes or hypertension^[5,36]^. It has been noted that one flaw of RCTs is unlikely to identify rare and delayed adverse effects (AEs), if any, in the real medical world. Several have postulated the need for mindful post-market surveillance^[3]^. In conjunction with this call for post-market, surveillance multidisciplinary sciences and active pharmacovigilance (PV) have emerged to detect, assess, understand and prevention of drug safety problems. In particular PV has emerged as a useful approach to expose rare and late occurring AEs during post-marketing surveillance with the assistance of artificial intelligence-facilitated RWD^[4,6]^. Certain PV assessment reports of post-approval monitoring across quite large populations have made it possible to update some drug labels with extra-safety warnings^[17]^. The latter will accordingly be helpful for relevant medical service to improve the clinical outcome with this medication through mindful tackling potential risks of the severe adverse event.

The advent of immunotherapeutics in oncology has improved clinical outcomes for patients with a wide variety of cancer. This is, at least in part, due to the success of immune check-point blocking antibodies including anti-cytotoxic T-lymphocyte antigen 4, anti-programmed cell death 1 (PD-1) receptor, and its ligand PD-L1^[37]^. However, these antibodies also generate a spectrum of immune related adverse events (irAEs), some of which are severe or very rare (frequency <1%), and are not revealed through the standard procedures of RCTs, particularly in those with accelerated approval ^[5–6]^. By taking advantage of various large-scale PV reporting of RWD, it has been proven that the profile of irAEs can be more comprehensively characterized. This has been the case for those receiving interventions to manage diabetes, multiple sclerosis, myocarditis, among others^[6]^. Coincidentally, through an insulin pathway-independent mechanism of action sodium glucose co-transporter-2 inhibitors (SGLT2i) has been found to confer a dramatic anti-diabetic effect with diminishing glucose and sodium absorption from the renal tubule. This has also by way of RWS been linked to minimizing the risks of heart failure and chronic kidney diseases^[5,17]^. However, enhanced glucosuria by SGLT2i in this case may predispose the patients to urogenital tract infection, implicating a type of exceptional adverse events of these medications.

Another example of where RWS has advanced our knowledge for clinical practice is Fournier gangrene (FG). FG is a rare but serious AE and was identified based on relevant RWD derived from super large-scale PV reporting systems for over 5 years. This led the US FDA to release a safety warning for SGLT2i and has generated further research into the causal pathways and into management techniques^[17]^. This is becoming more commonplace as RWS comes to the fore. For example, the cyclooxygenase-2 inhibitor, Rofecoxib, was withdrawn from the market due to the increased risk of myocardial infarction (MI). Rofecoxib was originally approved for treating arthritis although, the standard RCT approach ensured that those suffering underlying cardiovascular conditions were excluded^[18,35]^. Later, it emerged through RWD that Rofecoxib actually increased the risk of acute MI and had to be withdrawn because of the prevalence of comorbid MI^[18]^.

## Re-purposing therapeutic indications

There are a number of unmet clinical needs and there is a need to improve productivity in pharmaceutical innovation. These unmet needs could be realized by way of drug re-purposing which is a strategy to identify distinct or expanded indications for existing medications^[5,12]^. While minimizing the risks associated with pharmaceutics research and development, the re-purposing approach can bypass pre-clinical investigations and even can see studies beginning at a later phase in RCT procedures. This provides a number of advantages because the safety profile and efficacy may already have been established thereby reducing the time required to market and the finance implications^[38]^. Being inspired by either a pre-conceived hypothesis or incidental findings, drug-repurposing can provide a range of potential successes, some of which have been identified through RWS^[5,38]^. In particular, going beyond that of networked pharmacology or animal modelling, drug repurposing development based upon RWD can potentially deliver the practical information regarding effectiveness for a target population. This is particularly relevant to dose specificity and determining the route of administration and labelling the product for primary indications^[38]^.

For example, Heparin has served as a mainstream anticoagulant for nearly a century, and is used to prevent or to treat thrombotic events in various fields including coagulating complications in the patients with COVID-19^[19]^. Recently, a RWD-based retrospective study found that low molecular weight heparin played an additive role in anti-inflammation by attenuating the cytokine storm upon associated with IL-6 down-regulation^[20]^. Likewise, in the field of metabolic disease, glucagon-like peptide-1 receptor agonists (GLP-1RAs) have been noted to induce insulin-secreting cell activity, and thereby control blood glucose levels in type 2 diabetes^[39]^. Additionally, RWS-based research have found GLP-1RAs are also characterized as having a potential beneficial effect non-alcoholic fatty liver disease with the mechanisms of improving liver function and diminishing fibrosis in the patients^[21]^. Besides, molecular genetics analysis of real-world clinical data has surprisingly identified the Janus kinases/signal transducer and activator of transcription pathway to be a therapeutic target of Alzheimer's disease, which has been further validated by animal modelling^[22]^, although further clinical research is required.

## Biomarker validation

Over the past two decades, there have been a number of dramatic breakthroughs in life science which have revolutionized our understanding of numerous aspects of disease biology. These breakthroughs have helped to transform pharmaceutical innovations and to develop clinical care^[5,12]^. Accordingly, precision medicine has risen to improve the therapeutic outcomes by targeting a particular subset of patients or even a selective stage of disease, in which biomarkers are emerging as a valuable tool^[12,40]^. Of note, quantifying relevant biomarkers related to specific pathologies can offer more objective evidence than traditional physicals examinations^[40–41]^. In certain RCT scenarios, specific biomarker assays have been adopted and developed into a companion diagnostic kit along with innovative medications to maximize objective response rates in a sub-set of the disease population^[5]^. Moreover, the expanded utility of biomarker-based strategies have recently been revealed through RWS, which include serving as surrogates to reflect therapeutic outcomes or to detect early signals of pathogenesis for timely intervention^[41]^.

The aberrant expression of epithelial growth factor receptor (EGFR) is well-documented as a target for anti-cancer management. EGFR is also a biomarker for monitoring effectiveness of the relevant medications although evidence is limited^[42]^. A retrospective analysis of RWD covering over 6000 cases has shown that genomic profiling of circulating tumor DNA (ctDNA) is highly consistent with that of tumor tissue DNA. This can be applied to guide therapeutic options and has proven reliable for diseases such as non-small cell lung cancer^[23]^. On the other hand, to further delineate associations of outstanding biomarkers with the outcomes of heart failure (HF), a RWD-based study was conducted. It demonstrated that a diminished ejection fraction (EF) or/and elevated B-type natriuretic peptide (BNP) significantly correlated with an increased risk of stroke and acute MI in the patients with HF. This suggests that BNP and EF may serve as surrogates when considering the preventive measures^[24]^. In addition, the recent COVID-19 pandemic highlighted unmet clinical needs for biomarker analysis in sepsis development. Accordingly, a retrospective RWS has identified that the serial biomarkers of procalcitonin and C-reactive protein can be utilized to facilitate improvement in clinical outcomes for critically ill patients with sepsis^[25]^.

## Evaluation of economic benefits

To achieve projected clinical effectiveness, following approval of regulatory authorities both innovative and generic drugs need to evolve into established products accepted by public or private payers, and then more conveniently to reach patients^[4]^. The results of RCTs can provide the principle profile of relevant medical sciences for covering decision-making of payers^[1,5]^. Besides observing head-to-head comparisons, long-term effectiveness and safety in routine healthcare settings, RWD also address the realistic concerns such as patient compliance and medical costs^[4,43]^. From the perspective of patients and payers, while the effectiveness versus risks of a coming medication must be assessed clearly, reasonable costs or economic benefits compared to those of exiting standard of therapy for the same indication can not be ignored^[43–44]^. Eventually, only drugs with preferable benefit/risk margin and cost advantages will survive in real-world medical care systems in the long term^[44]^.

Anti-diabetic management represents a highly complex and competitive pharmaceutic market landscape with increasing options in recent years, which poses a number of challenges for clinicians and payers. A retrospective RWE-based RWS has identified the fixed-ratio combination insulin degludec/liraglutide for six months and are able to lower the incidence of diabetes-related complications and enhance quality-adjusted life expectancy while substantially reducing direct medical costs, compared to those of basal insulin, GLP-1 receptor agonists and oral medications^[26]^. In the anti-viral field, insightful into combinations of various retroviral inhibitors has significantly improved the clinical outcomes of patients with human immunodeficiency virus (HIV) infection. HIV creates a huge financial burden for both individuals and the health system. Generic versions of Lamivudine, Abacavir, and Efavirenz have recently seen a saving in medicals costs of 25% according to a RWD-driven investigation^[27]^. Having transformed certain advanced cancer into manageable chronic diseases, the success of antibody therapy has also added to the costs for healthcare systems. To make it more affordable, real-world health economic analyses indicate that application of biosimilars could save from 4.9 to 120 million Euros for each antibody treatment including Rituximab and Trastuzumab^[28]^.

## Delineation of clinical epidemiology

By characterizing etiology-related population and applying statistical analysis, clinical epidemiology draws a greater picture of medical science for the disease of interest, which in turn inspires diagnostic/therapeutic design, practice and post-market surveillance^[45–46]^. From this perspective, the roles of traditional RCTs appear to be limited due to restricted participant numbers and processing duration^[3]^. This is particularly the case for big data, which covers gene profiling, biological bank, electronic health care resources, and unprecedented pandemic, *etc*^[45,47]^. To deal with these trends, while certain adaptive clinical trial modes have been designed and implemented through modifying RCTs, RWE has substantially expanded taking the advantages of upcoming real world bigdata^[5,47]^. Consequently, a wide variety of contemporary RWS have been conducted to fill the critical information gaps in clinical epidemiology. These include co-morbidities and co-pharmacy in pathogenesis, as well as the most appropriate sampling time points for diagnostic detecting assay and emerging drug-drug interactions^[4,45,47]^.

In this regard, a RWD-based epidemiological investigation revealed that pre-existing chronic illness, such as hypertension and diabetes, substantially increases the mortality risk of SARS-CoV-2 infection^[22]^. To address a concern related to angiotensin-converting enzyme 2 (ACE2)-mediated SARS-CoV-2 invading, the RWE has demonstrated that antihypertensive drugs targeting the renin–angiotensin–aldosterone system do not raise the death rate of patients with the viral infection^[36,40]^. Moreover, a RWD-based clinical epidemiological study identified the most efficient quarantine duration and timing of diagnostic testing for COVID-19, which determined that the duration of a 14-day quarantine could be shortened by 50% with testing on exit^[41]^. Of note, a retrospective RWS revealed that 68% of the patients with COVID-19 were suffering from at least one potential drug drug interaction (DDI) and the risk of cardiotoxicity was increased upon the majority of severe DDIs, which signal a warning that polypharmacy-induced adverse effects must be carefully assessed prior to treating complicated disease like this^[4,42]^.

## Perspective

While serving as the gold standard for regulatory agencies to assess the safety and efficacy of potential interventions prior to market authorization, RCTs cannot confer all the solutions to judge pharmaceutical products due to limited trial population and duration^[1–2,5]^. RWE and RWS are utilized to play a pivotal complementary role in a range of aspects covering effectiveness corroboration, rare adverse event discovery, pharmaco-economic evaluation, *etc*^[2–3]^. Nonetheless, it should be realized that RWE also has its own challenging issues resulting from the dependence on the quality of highly heterogeneous RWD. The latter may have incompleteness, inaccuracy, inconsistency, and complicated confounders, which can be better solved through the RCT framework^[4,7]^. Realistically, while RWE is valuable in post-market safety surveillance^[3]^, RWD-based effectiveness validation may sometimes be misled by inappropriate use of the data information^[48]^. To mitigate the flaws of RWE, bias-associated RWD needs to be carefully processed through several evolving mechanisms including appropriate database, multi-variable matching/adjustment, robustness test and sophisticated statistical analysis^[4,49]^. In recent years, taking the advantage of rising modern techniques such as large-scale biological data harvesting (biobanks) and electronic consent/data linkages, RWS and RWE have been significantly facilitated in terms of improving efficiency and lowering trial costs/site burden^[47,50]^.

In the contemporary landscape of bio-medical innovation, RWE and RWS are going beyond the traditional roles of post-market surveillance, to exceptionally contribute to accelerated regulatory approval of certain breakthrough pharmaceutical products that address the significant unmet clinical needs^[12,51]^. While open-label, single-arm clinical trials were frequently designed to examine chimeric antigen receptor T-cell (CAR-T) therapies against advanced B-cell leukemia/lymphoma^[37]^, the comparable RWD of refractory myeloma have been prepared as a historical control for developing anti-myeloma CAR-T medicine^[52]^. In parallel, RWE approach has been utilized for the initial approval of a novel immune check point inhibitor Avelumab for treating metastatic Merkel cell carcinoma, which represents a rare aggressive disease with unmet medical need and very small patient population being unpractical to run a large RCT^[53]^. Well-managed RWD have been playing an unique role in the clinical evaluation of COVID-19 vaccines regarding extrapolation of population segments, duration of protection and safety monitoring^[51]^. Several officially channeled RWE plans have been integrated with RCT results into a presentation to the regulatory agency, successfully applying for an emergency use authorization of a novel COVID-19 vaccine^[51,54]^. It is fortunately trending that cutting-edge innovative medicine can be developed and delivered much more efficiently upon appreciating greater contributions from real-world clinical investigations with regulatory grade quality.
